# Allergic bronchopulmonary aspergillosis as an initial manifestation of cystic fibrosis: Diagnostic and therapeutic implications in the era of CFTR modulators

**DOI:** 10.1016/j.jacig.2024.100294

**Published:** 2024-06-27

**Authors:** Georgia Mitropoulou, Zisis Balmpouzis, Aurelia Oberhansli-Wavre, Michael Morris, Sylvain Blanchon, Alain Sauty, Angela Koutsokera

**Affiliations:** a Adult Cystic Fibrosis and CFTR-related disorders Center, Division of Pulmonology, Department of Medicine, Lausanne University Hospital and University of Lausanne, Lausanne, Switzerland; b Lung Transplant Center, Division of Pulmonology, Department of Medicine, Lausanne University Hospital and University of Lausanne, Lausanne, Switzerland; f Paediatric Pulmonology and Cystic Fibrosis Unit, Division of Paediatrics, Department of Woman-Mother-Child, Lausanne University Hospital and University of Lausanne, Lausanne, Switzerland; c Centre de pneumologie Bulle Santé, Bulle, Switzerland; d Rehabilitation Center, Fribourg Hospital, Riaz, Switzerland; e SYNLAB Genetics, Lausanne, Switzerland; g Division of Pulmonology, Department of Medicine, Neuchâtel Hospital Network, Neuchâtel, Switzerland

**Keywords:** Allergic bronchopulmonary aspergillosis, ABPA, cystic fibrosis, CFTR modulators

## Abstract

Allergic bronchopulmonary aspergillosis (ABPA) results from complex hypersensitivity reactions to *Aspergillus fumigatus*, which often occur in patients with asthma, cystic fibrosis (CF), or CF transmembrane conductance regulator (CFTR)-related disorders. Genetic predisposition, particularly variants of the *CFTR* gene, probably plays a significant role in the development of ABPA. We present the case of a 20-year-old male with ABPA and bronchiectasis that was initially misdiagnosed as a result of normal sweat chloride values and negative first-level genetic testing results. Comprehensive *CFTR* gene sequencing revealed 2 pathogenic variants, R347H and D1152H, which together with the clinical phenotype and functional tests, supported the diagnosis of CF. Treatment with elexacaftor/tezacaftor/ivacaftor resulted in significant clinical and functional improvement, including a marked decrease in total IgE levels, suggesting a potential role for CFTR modulators in controlling ABPA. This case illustrates the evolving understanding of CF as a spectrum of disorders in which CFTR dysfunction may manifest subtly and variably, necessitating a high index of suspicion and a comprehensive diagnostic approach to ensure timely treatment in the era of highly effective CFTR modulators.

Allergic bronchopulmonary aspergillosis (ABPA) results from complex hypersensitivity reactions targeted against *Aspergillus fumigatus* in susceptible patients. ABPA has been typically described in individuals with asthma or cystic fibrosis (CF) and may be the sole manifestation of a CF transmembrane conductance regulator (CFTR)-related disorder (CFTR-RD). In patients with ABPA who do not fulfil the diagnostic criteria of CF or CFTR-RD, genetic predisposition seems to play a significant role, as suggested by the increased frequency of variants of the *CFTR* gene in these patients versus in the general population.^1^

CF care has been transformed with the arrival of CFTR modulators, which are small molecules that improve the quantity and/or function of the CFTR protein. Modulator therapy, which is available today for approximately 85% of people with CF, has led to significant improvements in lung function and respiratory symptoms, as well as to a drastic reduction of respiratory exacerbations. Given the important implications of early access to highly effective modulator therapy (HEMT), timely and accurate diagnosis of CF is essential.

We present the case of an adult patient with ABPA for whom the diagnosis of CF was initially dismissed on the basis of a sweat chloride test result below the diagnostic threshold for CF and a negative result of first-level genetic testing. A more extensive workup led to the diagnosis of CF, allowing treatment with HEMT, which resulted in significant clinical and functional improvement.

## Case presentation

A 20-year-old male with allergic asthma and bronchiectasis complicated by haemoptysis had a prior workup, the results of which were diagnostic for ABPA based on increased levels of total and *Aspergillus*-specific IgE and *A fumigatus* precipitins, as well as serum eosinophilia. Besides *A fumigatus*, the patient was allergic to house dust mite, dog dander, and mugwort. The initial etiologic investigations of bronchiectasis did not allow establishment of an alternative diagnosis. More specifically, following 1 indeterminate sweat chloride test result [51 mmol/L (normal value, <30 mmol/L; value indicative of CF, ≥60 mmol/L)], the result of a repeat test was within normal range (28 mmol/L). The results of first-level genetic testing of 35 common *CFTR* variants were negative. Despite normal nasal NO levels, nasal biopsies were performed to investigate for primary ciliary dyskinesia. High-resolution video microscopy revealed static cilia with incomplete beat; however, transmission electron microscopy showed a normal ciliary ultrastructure. The patient’s levels of IgG (and subclasses thereof), IgM, IgA, and α1-antitrypsin were normal. On the basis of these elements, the diagnosis of CF was dismissed in favor of ABPA-related bronchiectasis. The patient began receiving oral corticosteroids and antifungal therapy. After 3 months of antifungal treatment, he developed an episode of pancreatitis and his treatment was interrupted.

Because of repeated respiratory exacerbations over the next 12 months, the patient was referred to our CF center for further investigations and a management plan. A detailed history revealed that he was born at full term, with no neonatal respiratory complications nor family history of pulmonary disease. His father was of Chilean descent and his mother was of Swiss origin. Over the years, his only identified clinical manifestations were respiratory (chronic productive cough since the age of 2 years and periods of symptomatic exacerbations with haemoptysis) and a single episode of drug-induced pancreatitis. Spirometry showed moderate airway obstruction (FEV_1_ value of 2.64 L; 68% of predicted). A chest computed tomography (CT) scan revealed upper and middle lobe predominant bronchiectasis. Cultures of the patient’s sputum were positive for methicillin-susceptible *Staphylococcus aureus*. Ear, nose, and throat assessment showed sinusitis without polyposis. Biologic testing confirmed ABPA, but the patient declined a new course of corticosteroids and antifungals, as he was planning a 9-month trip abroad. The patient was pancreatic sufficient with no gastrointestinal symptoms, a body mass index of 22 kg/m^2^, normal fecal elastase level, and normal findings of magnetic resonance imaging of the pancreas. Semen analysis revealed azoospermia.

**Fig 1:**
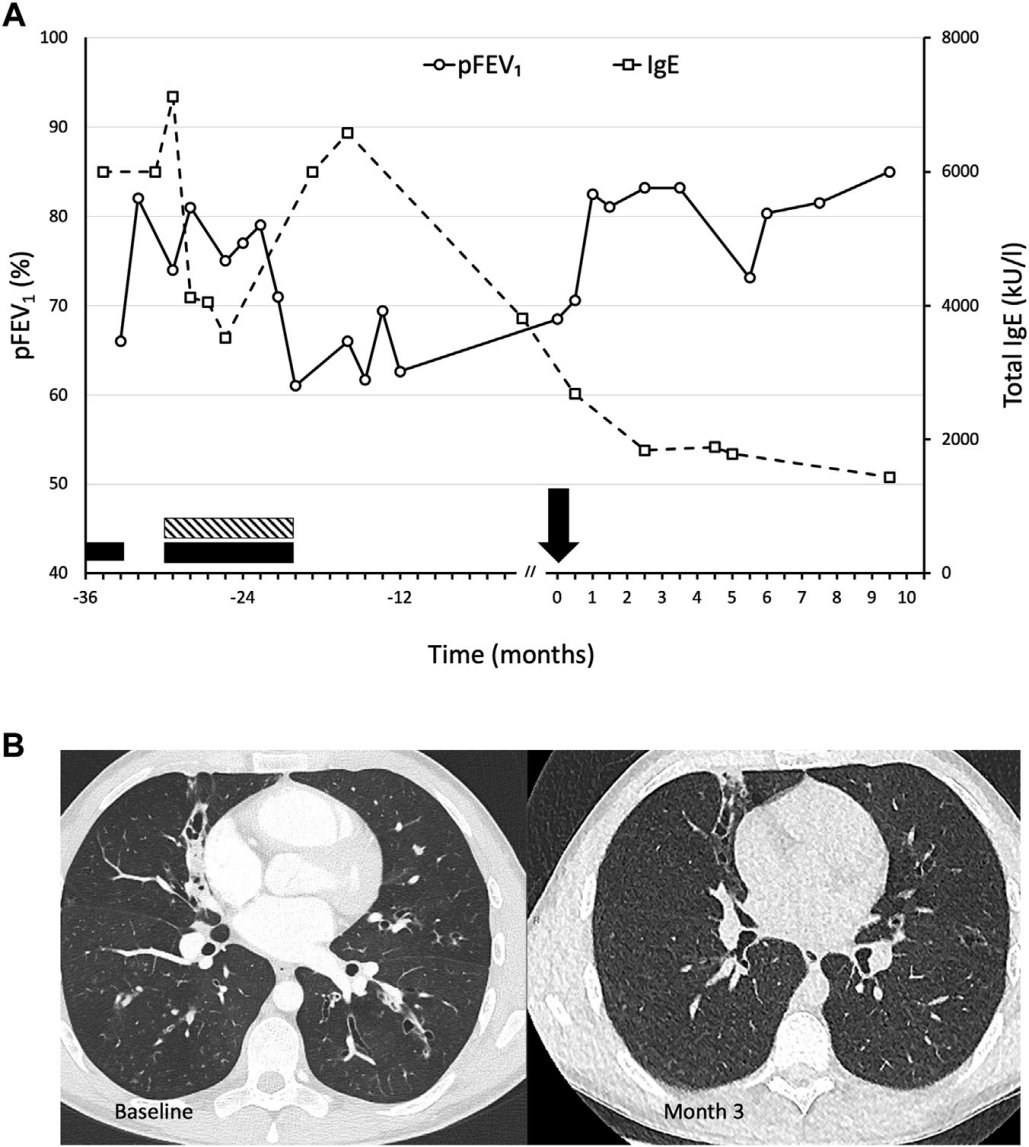
**A,** Evolution of lung function and IgE over time. The black rectangle indicates treatment with systemic corticosteroids, and the shaded rectangle indicates treatment with itraconazole. The black arrow indicates the start date of ETI administration. The patient did not receive corticosteroids or antifungals in either the 14 months preceding or the 10 months following initiation of ETI administration. **B**, Chest computed tomography scan 12 months before (baseline) and 3 months after treatment initiation shows a marked reduction in bronchial wall thickening, mucus plugging, and centrilobular nodules.

On the basis of these elements, the diagnosis of CF or CFTR-RD was revisited. The patient underwent a third sweat chloride test, the result of which was normal (26 mmol/L). Comprehensive sequence analysis of *CFTR* revealed 2 pathogenic variants: R347H and D1152H (*trans* phase confirmed by parental analysis). Both R347H and D1152H are class IV variants with varying clinical consequences that can be associated with CF or CFTR-RD and commonly result in sweat chloride levels less than 60 mmol/L. Nasal potential difference testing showed a borderline response to CFTR stimulation, which was suggestive of at least partial CFTR dysfunction following stimulation with isoprotenerol (a Δlow chloride/isoproterenol value of 6.5 mV [normal value, >6 mV]), whereas CF diagnostic scores were within abnormal range (Sermet score of 0.14 [normal score, >0.27]; Wilschanski index of 0.57 [normal value, <0.7]). The clinical phenotype, *CFTR* genotype, and potential difference testing results were considered supportive of CF despite the negative results of the sweat chloride test. As both identified variants were eligible for modulator therapy, the patient began receiving elexacaftor/tezacaftor/ivacaftor (ETI), after his return. Within a few days of treatment initiation, the patient’s respiratory symptoms improved significantly, and within 3 months, his FEV_1_ value increased by 15% (3.56 L; 83% of predicted) and his sweat chloride value decreased to 16 mmol/L. Repeat chest CT showed a marked decrease in bronchial wall thickening, mucus plugging, and centrilobular nodules. Following ETI initiation, the patient’s total IgE levels decreased from 3811 kU/L to 1787 kU/L, with neither systemic corticosteroid or antifungal therapy nor treatment with biologic agents (Fig 1).

## Discussion

CF is an autosomal recessive multisystem disease resulting from variants of the *CFTR* gene. Diagnosis typically relies on “classic” clinical manifestations associated with abnormal sweat chloride measurements (≥60 mmol/L verified twice) or the presence of 2 CF-causing variants in *trans*. The diagnosis of CF or CFTR-RD may be challenging, especially in adults, in whom standard diagnostic criteria are not clearly fulfilled, and when other diagnoses, such as asthma and ABPA, may overlap with CF/CFTR-RD. For instance, the presence of peripheral bronchiectasis in a patient with ABPA is strongly suggestive of a second underlying condition causing bronchiectasis.^2^ As shown in this case, some variants may be associated with a normal sweat chloride test result. Furthermore, in atypical cases and/or non-White individuals, first-level genetic testing may be insufficient for effective mutation detection.

Various mechanisms of immunogenetic susceptibility for ABPA, such as HLA-DR restriction and HLA-DQ protection and polymorphisms of the IL-4 receptor α-chain, IL-10, surfactant protein A2, and Toll-like receptors, have been suggested in populations with CF and populations without CF.^3^ However, it appears that there is a risk of ABPA directly related to defects of CFTR expression. A meta-analysis found an increased frequency of *CFTR* gene variants in patients with ABPA versus in the general population (odds ratio = 10.39 [95% CI = 4.35-24.79]).^4^ It is also known that CFTR is expressed on T cells and that the absence of CFTR in naive CD4^+^ T cells from CFTR-deficient mice leads to an excessive T2 inflammatory response to *A fumigatus*.^5^ Hence, it has been postulated that, in the presence of abnormal airway mucus secondary to CFTR dysfunction, exposure to *Aspergillus* allergens can result in heightened bronchoalveolar mucosal immune response and development of ABPA.^6^ Although *CFTR* variants have not been detected in all patients with ABPA, most studies have used limited genetic screening panels and not whole-gene analysis, thus potentially underdiagnosing patients with rare variants.^1^

The IgE levels of the patient in this case report decreased markedly following ETI initiation; however, on the basis of current data, this improvement cannot be attributed solely to HEMT. It is possible that the patient’s 9-month absence, and thus, his removal from his usual environment, contributed to the decrease in IgE levels from more than 6000 UI to 3811 UI, but this decrease was not associated with an improvement in lung function. Moreover, the patient’s total IgE counts had never before dropped below 3000 kU/L, even during ABPA treatment. A sustained decrease to levels less than 2000 kU/L and a prolonged improvement in lung function were observed only after the patient had begun receiving ETI. The role of CFTR modulators in the control of ABPA therefore remains to be elucidated; the preliminary evidence suggests a dampened *Aspergillus*-induced reactive oxygen species production by CF phagocytes in patients treated with ivacaftor alone or in combination with lumacaftor.^7^ In a recent retrospective study, treatment with ETI was associated with a significant decrease in the ratio of *Aspergillus* spp–positive sputum cultures, as well as with a reduction in levels of anti-*Aspergillus* precipitating antibodies and total IgE.^8^ Furthermore, recently published evidence suggests that initiation of HEMT is associated with a reduction in antigen-specific CD154^+^ T-cell proliferation activity, which is involved in aberrant immune responses with hyperinflammation, as in the case of ABPA.^9^

This case highlights the fact that in the era of highly effective CFTR modulators, a diagnosis of ABPA should prompt a comprehensive workup for CF. Recognizing the wide spectrum of CFTR dysfunction and being aware of diagnostic testing limitations are essential to avoid missing or delayed CF diagnosis.

## Disclosure statement

Supported by the 10.13039/100000001Swiss National Science Foundation (BRIDGE Discovery Grant 40B2-0_194701/1 [to A.K.]), Cystic Fibrosis Switzerland (research grant [to G.M.]), the Lung Association of the Canton of Vaud (research grant [to G.M.]).

Disclosure of potential conflict of interest: G. Mitropoulou reports honoraria from Vertex Pharmaceuticals. Z. Balmpouzis reports honoraria from Vertex Pharmaceuticals and OM Pharma. M. Morris is an employee of SYNLAB International GmbH. The rest of the authors declare that they have no relevant conflicts of interest.
